# ASO Author Reflections: Chemotherapy Regimen in Borderline Resectable and Locally Advanced Pancreatic Cancer—Resection Cuts the Deal

**DOI:** 10.1245/s10434-023-13484-6

**Published:** 2023-04-19

**Authors:** Dilmurodjon Eshmuminov, Botirjon Aminjonov, Russell F. Palm, Kuno Lehmann

**Affiliations:** 1 Department of Surgery and Transplantation, University Hospital Zurich, University of Zurich, Zurich, Switzerland; 2 Department of Radiation Oncology, H. Lee Moffitt Cancer Center and Research Institute, Tampa, FL USA

## Past

In patients with locally advanced (LAPC) or borderline resectable (BRPC) pancreatic cancer, primary chemotherapy with or without radiotherapy is recommended for local downstaging to enable a potentially curable resection. FOLFIRINOX or gemcitabine-based chemotherapy are in common use. However, it is currently unclear what chemotherapy should be preferred for patients with LAPC and BRPC. Previous studies investigating this question are available, but often include a limited number of patients, combine LAPC with BRPC, or combine both chemotherapy regimen.^1–4^

## Present

In this study, we report for the first time the outcomes separately for BRCP and LAPC, and by chemotherapy regimen including FOLFIRINOX or gemcitabine-based regimen with the largest patient number currently available from high-volume international centers (Eshmuminov et al. in press).^5^ Intriguingly, for resected patients, outcomes are similar between FOLFIRINOX or gemcitabine-based regimen for both BRPC and LAPC. Primary treatment with FOLFIRINOX compared with gemcitabine-based chemotherapy appears to provide a survival benefit for patients who are ultimately unresectable.

## Future

FOLFIRINOX is preferred for patients with good performance status due to the survival benefit in patients who ultimately do not undergo resection. Gemcitabine-based regimen can be considered as a reasonable alternative to FOLFIRINOX, particularly in patients who have a less robust performance status. Nevertheless, resection offers the best chance for prolonged survival. Future prospective studies are necessary to optimize patient selection for each regimen as well as clarify the optimal duration of neoadjuvant treatment.
